# Esophageal Intramural Haematoma related Dysphagia: A rare complication after thrombolysis

**DOI:** 10.34172/jcvtr.2022.11

**Published:** 2022-04-06

**Authors:** Samman Verma, Prashant Gupta, Amitava Dutta, Pankaj Gupta, Vishal Sharma

**Affiliations:** ^1^Department of Internal Medicine, Post Graduate Institute of Medical Education and Research, Chandigarh, India; ^2^Department of Gastroenterology, Post Graduate Institute of Medical Education and Research, Chandigarh, India; ^3^Department of Radiodiagnosis, Post Graduate Institute of Medical Education and Research, Chandigarh, India

**Keywords:** Dysphagia, Myocardial infarction, Anticoagulant, Hematemesis, Thrombolysis, Streptokinase

## Abstract

Esophageal Intramural Haematoma (EIH) is a rare entity usually caused by repeated emesis or trauma. It is diagnosed on the basis of upper gastrointestinal endoscopy and radiology. Treatment is conservative unless hemodynamic instability prevails. Use of anticoagulation or thrombolytic therapy is believed to be a risk factor rather than a causative etiology. However, a review of literature shows only few cases occurring post-thrombolysis. We report about a patient of myocardial infarction who was thrombolyzed with streptokinase. He developed hematemesis and dysphagia a few hours after thrombolysis despite ECG resolution of his ST elevation. He was diagnosed to have EIH on basis of endoscopic and computed tomographic findings. His symptoms improved within two weeks, and a repeat UGIE showed resolution of the hematoma.

## Introduction

 EIH is an uncommon entity, with a good prognosis. The clinical presentation consists of a triad of: Chest pain (84%), dysphagia/odynophagia(59%), and haematemesis (56%). ^1^ All features of the triad are present in around 1/3^rd^ of the patients, while around 70% have at least two symptoms.^2,3^

## Case Presentation

 A 63 year-old male presented to our emergency department with retrosternal chest pain for 5 hours. It was sudden in onset, with radiation to left shoulder. He had no known co-morbidities. He had history of occasional alcohol intake, and he was a chronic smoker, with a 10 pack-year history. On examination, his vitals were stable, and systemic examination was unremarkable. An Electrocardiogram (ECG) showed Anterior Wall ST segment Elevation Myocardial Infarction. He was thrombolyzed with Streptokinase (1.5 million units in 100 ml of dextrose over 60 minutes), and given intravenous heparin (3000 IU followed by 12 mg/kg infusion). Repeat ECG showed resolution of the ST segment Elevation. The patient was kept under observation. 12 hours after thrombolysis, he developed new onset retrosternal chest pain, associated with 1 episode of haematemesis, and dysphagia. The haematemesis comprised of only 60 mL of blood. There was no bleeding from any other site. The patient was hemodynamically stable and did not require blood transfusion. ECG was not suggestive of re-infarction.

**Figure 1:**
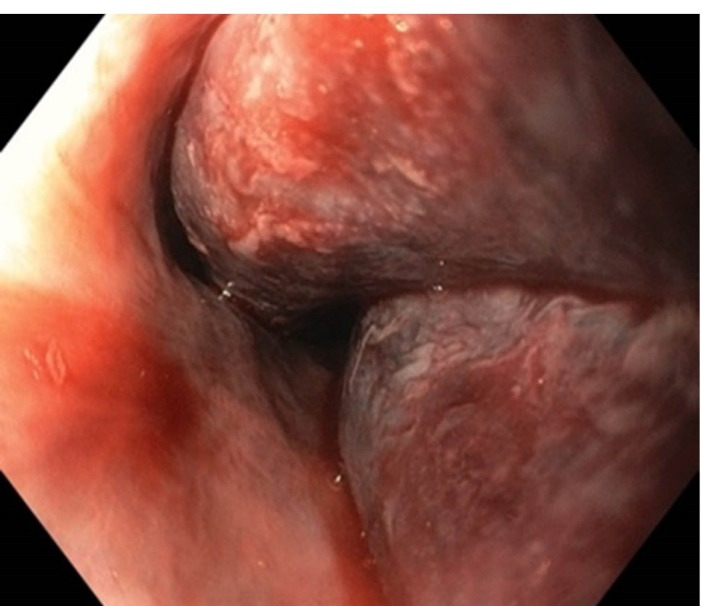
UGIE done showing a bluish discoloration in the posterior wall of the oesophagus suggestive of intramural haematoma

**Figure 2:**
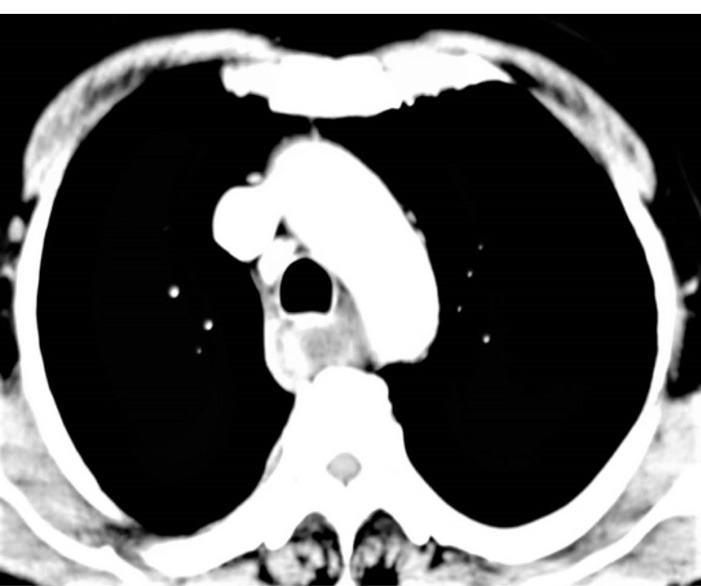
CECT Chest of the patient at the level of arch of aorta showing a dilated oesophagus with eccentric heterogeneously hypodense esophageal wall thickening suggestive of the hematoma

 The heparin was stopped. Aspirin was continued throughout but clopidogrel was stopped and added at the time of discharge After initial resuscitation, a UGIE was done, which showed a bluish discoloration in the wall of the esophagus, from middle to the lower part (Figure 1). There was no active bleed present.A Computed Tomography (CT) Angiography was done which was consistent with the diagnosis of EIH (Figure 2). The clinical diagnosis of EIH is important in ruling out other differentials, which may have worse prognosis.

**Figure 3:**
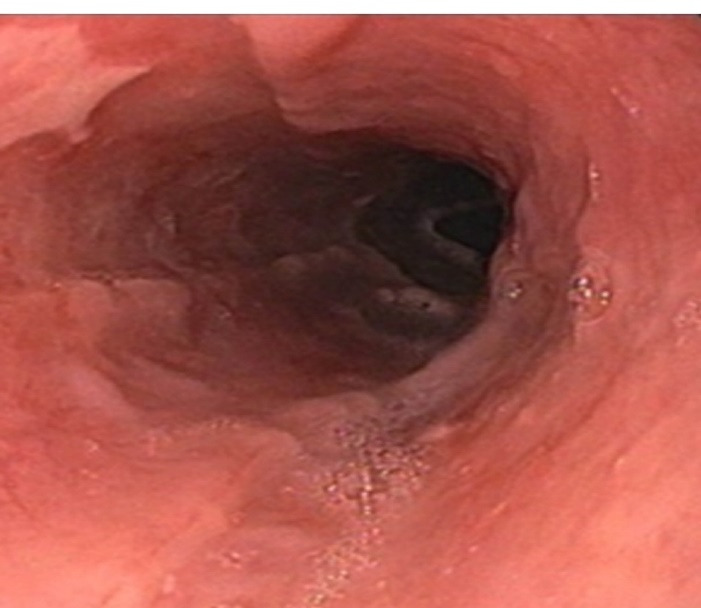
Repeat UGIE done after 2 weeks showed complete resolution of the haematoma

 The patient was kept nil per oral for 2 days. He was started on IV Fluids and Proton Pump Inhibitors. Liquid followed by semisolid food was slowly reintroduced over the course of the following days. The patient was monitored closely for any further deterioration. His symptoms abated. A repeat Upper GI Endoscopy (Figure 3) done after 2 weeks showed complete resolution of the haematoma.

## Discussion

 The largest review series on EIH, of 174 cases, showed a bimodal distribution, with one peak at 45 years, and one at about 70 years.^1^However, in recent literature, only a few cases have been reported where EIH has occurred in the setting of thrombolysis. All 3 have occurred in different settings of thrombolysis, vis a vis for acute ischemic stroke, pulmonary thromboembolism, and myocardial infarction.^4-6^The thrombolytic agents used in these reports were intravenous alteplase (0.9 mg/kg) and Reteplase, while the agent was not mentioned in one of the report. Our case also adds to evidence for the same. Chest pain, especially in the setting of thrombolysis post myocardial infarct, could indicate re-infarction, or complications like ventricular aneurysmal or free wall rupture. However, a repeat ECG and Echocardiogram were not suggestive of the same. Aortic dissection, though unlikely, was also ruled out by the CT scan. In view of haematemesis and dysphagia, esophageal causes like Mallory-Weiss tear and Boerhaave syndrome were suspected in our patient. The same were ruled out by both a CT scan and Upper Gastro-Intestinal Endoscopy.

 While the benefit of thrombolysis has been well established by the Fibrinolytic Therapy Trialists’ Collaborative Group, it is still wrought with its own complications, chiefly, bleeding. An analysis of the GUSTO I trial showed that 11.4% of patients undergoing thrombolysis for myocardial infarction developed at least moderate haemorrhage at various sites.^7^ The principal risk factors for bleeding were: Increased age, low weight, female sex, African ancestry, and invasive procedures. Use of streptokinase also made the patient more prone to bleeding complications than tPA. The analysis further showed that gastrointestinal bleed comprised 1.5% of moderate, and 0.3% of severe bleed complications.

 The diagnosis of EIH is established by endoscopy and radiology. In Upper GI Endoscopy, the haematoma is seen as a bluish discoloration located most commonly in the lower part of the esophagus as it is deficient in striated muscles and other supporting structures.^2^ Radiological investigations include CECT, Magnetic Resonance Imaging (MRI), and CT/MRI Angiography. The typical appearance on CT is of an eccentric hyperattenuating mass in the wall of the esophagus.^8^ MRI may be used to better delineate the soft tissue planes.

 The prognosis of EIH is usually good, with around 75% recovering in around 2 weeks.^1^ There are no established guidelines for management. Treatment is usually conservative. It involves keeping the patient nil per oral for a few days, providing IV supplementation, and proton pump inhibitors. Food may be gradually re-introduced. Antibiotics are indicated only if an infection is suspected. Surgery may be considered for ongoing bleed, perforation or an abscess.

 The American College of Cardiology 2017 Expert Consensus Decision Pathway can be used to decide on stopping and restarting antiplatelet therapy, which may be an important decision in the setting of myocardial infarction.^9^

## Conclusion

 Esophageal Intramural Haematoma is a rare disease, which must be considered in the setting of chest pain, haematemesis, and dysphagia or odynophagia in the appropriate clinical setting. Management is mostly conservative, with a good prognosis overall.

## Funding

 None.

## Competing interest

 None.
